# Early Socio-Emotional Difficulty as a Childhood Barrier to the Expected Benefits of Active Play: Associated Risks for School Engagement in Adolescence

**DOI:** 10.3390/ijerph21101353

**Published:** 2024-10-13

**Authors:** Laurie-Anne Kosak, Kianoush Harandian, Simon L. Bacon, Isabelle Archambault, Luca Correale, Linda S. Pagani

**Affiliations:** 1 School of Psycho-Education, University of Montreal, Montreal, QC H2V 2S9, Canada; laurie-anne.kosak@umontreal.ca (L.-A.K.); kianoush.harandian@umontreal.ca (K.H.); isabelle.archambault@umontreal.ca (I.A.); 2 School Environment Research Group, University of Montreal, Montreal, QC H3C 3J7, Canada; 3 Montreal Behavioural Medicine Centre (MBMC), Centre Intégré Universitaire de Santé et de Services Sociaux du Nord-de-l’Île-de-Montréal (CIUSSS-NIM), Montreal, QC H4J 1C5, Canada; simon.bacon@concordia.ca; 4 Department of Health, Kinesiology and Applied Physiology, Concordia University, Montreal, QC H4B 1R6, Canada; 5 Sports Science Unit, Department of Public Health, Experimental and Forensic Medicine, University of Pavia, 27100 Pavia, Italy; luca.correale@unipv.it; 6 Sainte-Justine’s Hospital Research Center, Montreal, QC H3T 1C5, Canada

**Keywords:** active play, physical activity, school engagement, child development, longitudinal analyses

## Abstract

Active play allows children to develop social and cognitive skills, which could lead to higher school engagement. Little is known about the role of child socioemotional difficulty in these associations. This study aims to examine the interaction between active play and socioemotional difficulty in childhood and their prospective association with academic engagement in adolescence. The participants were 4537 children (51.1% boys) who were longitudinally followed, between ages 6 and 14 years, from the National Longitudinal Study on Children and Youth (NLSCY), Canada. Active play (weekly organized sport and unstructured physical activity outside of school hours) and child behavior (hyperactivity, anxiety, and relational difficulties) were reported by mothers for their children at age 6 years. Academic engagement was self-reported at age 14 years. Unstructured physical activity predicted lower subsequent school engagement for boys (β = −0.057, *p* < 0.05). Boys with high anxiety symptoms and high relational aggression who participated in more unstructured physical activity in childhood were subsequently less engaged in school (respectively, β = −0.066, *p* < 0.05 and β = −0.062, *p* < 0.05). Girls who partook in more organized sports showed lower school engagement in adolescence when they had high anxiety symptoms (β = −0.067, *p* < 0.05). Although past studies have highlighted the contribution of active play to school engagement, certain socioemotional difficulties could impede the child’s ability to reap its benefits.

## 1. Introduction

Early childhood represents a crucial time in developing lifelong habits [1]. Habits such as physical activity have decreased in children in recent years, especially outdoor play [2,3]. Given that humans are creatures of habit, active leisure has been given low priority. Barriers to active play, such as financial difficulties, lack of time, lack of access to play spaces, and safety concerns, are partly responsible for this low involvement [4,5]. However, the perceived importance of active play has been complexified by technological advancement in recent years [6]. This has led to a decrease in active play, which is disconcerting, considering that children who partake in moderate to vigorous physical activity for less than one hour per day have less optimal physical health and more behavioral difficulties [7].

Active play can be defined as a pleasant, creative activity that leads to child energy expenditure in body movement and brain activity [8]. It engrosses physical activity, which is most often present in the form of play for preschoolers [9]. Active play is a key contributor to healthy development. It allows children to expend more energy and to improve their motor skills [9,10]. This can lead to better cognition, language, and social interactions for children [11]. Childhood active play likely facilitates skill development through effortful participation. This might forecast long-term work and play habits that comprise lifestyle. For instance, children who practice more physical activity might have higher school engagement, which is defined as the level of commitment and participation in school and in academic tasks from a student, especially in adolescence [12,13].

Positive youth development theory suggests that practicing group sports fosters healthy involvement by promoting social interaction, confidence, and leadership [14]. Children may develop these skills by participating in physical activity, where socioemotional and cognitive skills, empathy, self-control, self-worth, and fair play are key [15]. Having an adult present as a teacher and a role model might also help youth develop these skills [15]. Having positive social interactions and better self-esteem are related to higher school engagement, mostly because they affect perceived student–teacher and peer relations [16,17,18].

Some behavioral dispositions may interfere with child active play and student engagement. Some child characteristics are notable when parents, acting as the primary decision-makers for preschool-aged children, promote active play and enroll their children in sports. In fact, parents tend to promote physical activity when a child is already active [19]. Thus, parents of hyperactive children might encourage active play more than in neurotypical children. Hyperactive and inattentive children singularly benefit from exercise as it has a positive effect on cognition, behavior, and physical health [20]. Since these children tend to have lower school engagement, this could contribute to their commitment and participation in school in a positive way [21]. 

On the other hand, children who are more anxious might partake in less physical activity, although this has yet to be verified. In a meta-analysis that looked at physical activity, sedentary behavior, and mental health, Rodriguez-Allyon et al. [22] found that no studies on these relations in preschoolers exist. However, it is known that anxious adolescents are less likely to partake in sports because of their low self-competence and that practicing less physical activity can lead to an increase in anxiety symptoms [23,24]. The emotional dysregulation felt by children with high anxiety might also interfere with learning processes, resulting in lower cognitive and affective school engagement [25].

Young children can resort to relational aggression, intentionally hurting someone by manipulating relationships [26] when they have not yet learned how to manage their emotions in a socially acceptable manner [27]. Active play in childhood fosters better expressive and conforming social skills [28]. When these skills are lacking, relational aggression can lead to student–teacher conflict in childhood, which could hinder school engagement [26].

Longitudinal research on the relationship between sport and achievement presents many limits, as it has primarily focused on academic performance rather than on task orientation. Moreover, little research has looked at the possible impact of early socioemotional difficulty on these associations [22]. Symptoms of hyperactivity/inattention, anxiety, and relational difficulties could have repercussions on academic engagement and learning [25]. Finally, not only are boys and girls different biologically, but they also experience active play and school engagement differently. Boys are often enrolled in sporting activities by their parents at a younger age than girls, whereas girls are directed towards less physical hobbies [29]. On the other hand, girls tend to be more engaged in school than boys [30]. Therefore, it is important to treat them as distinct populations when looking at variables relating to their development. This study aims to address these gaps by examining socioemotional difficulty and using a sex-stratified approach.

The purpose of this study is to examine the longitudinal associations between childhood active play and school engagement in adolescence. We also investigate the possible influences of behavioral disposition on these associations. More specifically, we examine whether the presence of socioemotional difficulty at age 6 years moderates the association between active play at age 6 years and later school engagement at age 14 years. We hypothesize that higher levels of childhood anxiety and relational aggression will impede the benefits associated with active play, whereas these benefits will be emphasized in those with higher hyperactivity-inattention symptoms.

## 2. Materials and Methods

### 2.1. Participants

The participants are from the National Longitudinal Survey of Canadian Children and Youth (NLSCY). The NLSCY is a nationwide longitudinal dataset using a sequential multiple cohort design that was developed by Human Resources Development Canada and Statistics Canada (https://www23.statcan.gc.ca/imdb/p2SV.pl?Function=getSurvey&Id=3513, accessed on 29 August 2024). Cycle 1 data were collected in 1994–1995 from a randomly selected birth cohort and yielded 22,831 participants aged 0 to 11 years. The selected children were followed biennially, and more participants, aged 0 to 2 years, were added upon each cycle. Residents living on Indian reserves, Crown lands, institutions, remote regions, and full-time members of the Canadian Armed Forces were excluded from the survey. Data were collected via computer-assisted interviews with the person most knowledgeable (PMK) of the child. Paper questionnaires were distributed to the children aged 11 years and older. In most cases (88.9%), the biological mother served as the PMK. Data were collected over a 9-month period for each cycle.

The participants for this study are a subsample of 4537 children (51.1% boys) who were 6 years old at Cycle 1, Cycle 2, and Cycle 3 and had available data on active play. The data were pooled on selected variables. Data from the first time point of the study were collected between 1994 and 1999. 

### 2.2. Measures 

#### 2.2.1. Childhood Active Play Predictors (Age 6 Years)

The PMKs reported on child active play at age 6 years on 2 items regarding organized sport and unstructured physical activity, respectively: “In the last 12 months, outside of school hours, how often has your child: (a) Played sports with a coach or instructor, other than in gym class? (school teams, swimming lessons, etc.)? (b) Played sports or done physical activities without a coach or an instructor (biking, skateboarding, etc.)?”. The responses ranged from 1 = almost never, 2 = about once a month, 3 = about once a week, 4 = a few times a week, and 5 = most days.

#### 2.2.2. Socioemotional Moderators (Age 6 Years)

For the following variables, scores were computed as 0 = below one standard deviation above the mean and 1 = above one standard deviation above the mean/at risk. See Appendix A for more details.

*Hyperactive-inattentive behavior*. The PMKs completed an 8-item scale (α = 0.84) regarding hyperactive and inattentive behavior.

*Anxious behavior*. The PMKs completed an 8-item scale (α = 0.79) regarding anxious behavior. 

*Relational aggression*. The PMKs completed a 5-item scale (α = 0.78) regarding relational aggressive behavior (Direct Indirect Aggression Scales [31]). 

#### 2.2.3. School Engagement Outcomes (Age 14 Years)

The youth completed a 6-item scale on behavioral, affective, and cognitive aspects of school engagement (α = 0.71 for girls; α = 0.68 for boys [32]). The sum of the scores forms a scale ranging from 6 (very low school engagement) to 18 (very high school engagement). See Appendix A for more details.

#### 2.2.4. Family Control Variables (Age 6 Years)

The details of the family control measures can be found in the Appendix A.

*Family configuration* (0 = intact family, 1 = non-intact family/at risk).

*Income adequacy* (0 = lower-middle, middle, upper-middle, or highest, 1 = lowest/at risk).

*Maternal education* (0 = graduated high school, 1 = did not graduate high school/at risk).

*Maternal depressive symptoms* were derived from the CES-D depression rating scale ([33], 0 = below one standard deviation above the mean and 1 = above one standard deviation above the mean/at risk).

*Family dysfunction* was derived from the McMaster Family Assessment Device ([34], 0 = below one standard deviation above the mean and 1 = above one standard deviation above the mean/at risk).

*Family social support* was derived from the Social Provisions Model ([35], 0 = above one standard deviation below the mean and 1 = below one standard deviation below the mean/at risk).

### 2.3. Data Analytic Strategies

The descriptive statistics and analyses were computed using SPSS (v. 28). Long-term prospective associations were estimated using ordinary least squares regression, stratified by sex. Youth school engagement at age 14 years was linearly regressed on the variables reflecting childhood active play. Adjustments for possible omitted variable bias were accounted for by including family characteristics that are either statistically or theoretically linked to the predictor or outcomes. Socioemotional behavior variables were used as moderators in the analyses [36,37]. For each outcome, the controls were entered, followed by the predictors to generate a fully controlled model, and then the moderator variable was entered to generate a model that included moderation for socioemotional issues at age 6 years. 

The analyses were weighted according to the weight assigned to each participant by Statistics Canada. The participants’ weight was calculated based on their design weight, non-response rate, and post-stratification. Thus, the analyses were adjusted to represent the entire population covered by the NLSCY.

#### Incomplete Data

This study required data from various sources and waves. As expected from a longitudinal study, some participants had incomplete data. Non-applicable and Does not know answers were coded as missing data. Data were missing at random. The attrition rate for the participants of this study was 37.1% between Time 1 and Time 2. Of the participants who had data on the predictor, 52.9% did not have complete data on the school engagement outcome. Girls with incomplete data at age 14 years were more likely to come from non-intact families (*X*^2^ (1, N = 2218) = 11.847, *p* < 0.001), to have a mother who did not graduate from high school (X^2^ (1, N = 2216) = 30.789, *p* < 0.001), and to have little family social support (*X*^2^ (1, N = 1535) = 9.011, *p* < 0.01). Boys with incomplete data at age 14 years were more likely to have a mother who did not graduate from high school (*X*^2^ (1, N = 2316) = 17.541, *p* < 0.001). We used multiple imputation in SPSS v.28 (IBM Statistics, Armonk, NY, United States: IBM Corp) to correct for attrition bias in the analyses.

## 3. Results

### 3.1. Descriptive Statistics

Table 1 reports the descriptive statistics for all variables of the study. Nearly half of boys and girls almost never partook in organized sport at age 6 years (41.5% and 52.7%, respectively). Moreover, 22.3% of the boys almost never engaged in unstructured physical activity, while 35.4% of them were doing so daily. For girls, most were never partaking in unstructured physical activity at age 6 years (28.9%), but 25.9% were doing so daily. Regarding socioemotional difficulty, 17.5% of the boys had high hyperactivity and inattention symptoms, 19.1% had high anxiety symptoms, and 13.3% had high relational aggression. For girls, 9.8% had high hyperactivity and inattention symptoms, 16.9% had high anxiety symptoms, and 16.5% had high relational aggression. At age 14 years, school engagement was high, with mean scores of 13.63 (SD = 1.82) for boys and 14.03 (SD = 1.87) for girls.

### 3.2. Associations between Family Characteristics, Socioemotional Difficulty, and Active Play at Age 6 Years

Table 2 reports the weighted unstandardized regression coefficients and standard errors reflecting the adjusted relationship between socioemotional difficulty and family characteristics at age 6 years, as well as active play at age 6 years, for boys and girls. Boys with high anxiety were partaking in less unstructured physical activity (β = −0.10, *p* < 0.001), whereas boys with high relational aggression were more likely to do so (β = 0.05, *p* < 0.05). Boys were engaging in less organized sports when their family was not intact (β = −0.13, *p* < 0.001), when their mother had not graduated from high school (β = −0.13, *p* < 0.001), and when their family had little social support (β = −0.10, *p* < 0.001). They were also practicing less unstructured physical activity when their family was not intact (β = −0.11, *p* < 0.001) and had low social support (β = −0.10, *p* < 0.001).

For girls, higher hyperactivity and inattention symptoms were associated with a decrease in unstructured physical activity (β = −0.07, *p* < 0.05), whereas higher anxiety symptoms were associated with an increase (β = 0.06, *p* < 0.05). Similarly to boys, coming from a non-intact family (β = −0.12, *p* < 0.001) and having a mother who did not graduate from high school (β = −0.11, *p* < 0.001) were associated with a lower participation in organized sport.

### 3.3. Direct Associations between Active Play at Age 6 Years and School Engagement at Age 14 Years

Table 3 reports the weighted unstandardized regression coefficients and standard errors reflecting the adjusted relationship between active play, socioemotional difficulty, and family characteristics at age 6 years and school engagement at age 14 years for boys and girls. Active play significantly predicted later school engagement for boys. An increase of 1 unit in unstructured physical activity at age 6 years predicted a 5.7% reduction in school engagement at age 14 years (*p* < 0.05, 95% CI: −0.122, −0.006). Non-intact family configuration and high family social support were also associated with a decrease in school engagement (respectively, β = −0.054, *p* < 0.05, 95% CI: −0.421, −0.004 and β = 0.070, *p* < 0.01, 95% CI: 0.110, 0.695).

No significant associations between active play at age 6 years and school engagement at age 14 years were found for girls. However, childhood socioemotional difficulties were associated with later school engagement. More specifically, high anxiety symptoms at age 6 years predicted a 7.7% SD unit decrease in adolescent school engagement (*p* < 0.01, 95% CI: −0.607, −0.101). School engagement was also reduced by having a non-intact family (β = −0.119, *p* < 0.001, 95% CI: −0.738, −0.279), a dysfunctional family (β = −0.063, *p* < 0.05, 95% CI: −0.660, −0.030), or a mother who did not graduate from high school (β = −0.078, *p* < 0.01, 95% CI: −0.637, −0.116).

### 3.4. Moderation Effect of Socioemotional Difficulty at Age 6 Years on the Associations between Active Play at Age 6 Years and School Engagement at Age 14 Years

**Figure 1:**
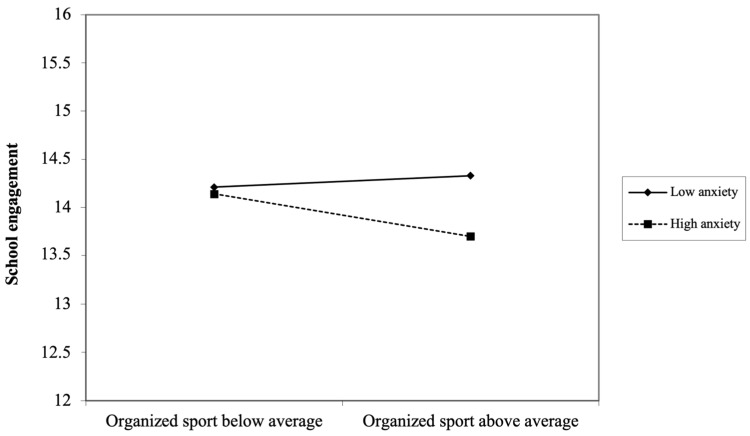
Decomposition of the adjusted interaction between anxiety symptoms and organized sports at age 6 years associated with school engagement at age 14 years for girls.

Socioemotional difficulties had a significant effect in moderating the associations between active play and later school engagement for all. Notably, for girls, anxiety symptoms stood out as a significant moderator. That is, when they experienced high anxiety symptoms in childhood, girls who partook in more organized sports at age 6 years were subsequently less engaged in school (β = −0.067, *p* < 0.05, 95% CI: −0.525, −0.029). Figure 1 illustrates the decomposition of this interaction.

**Figure 2:**
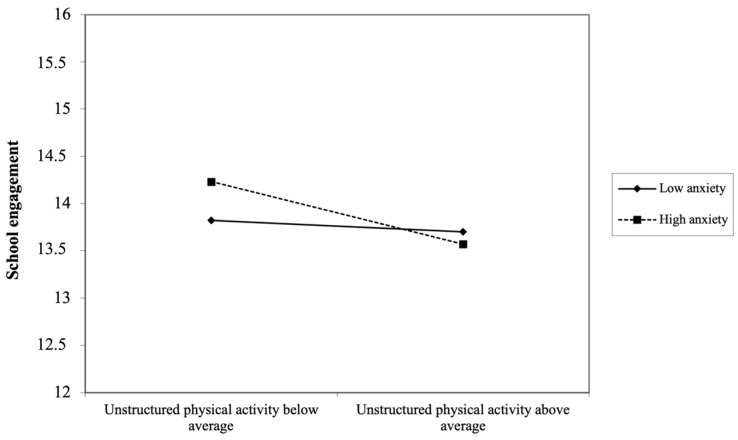
Decomposition of the adjusted interaction between anxiety symptoms and unstructured physical activity at age 6 years associated with school engagement at age 14 years for boys.

**Figure 3:**
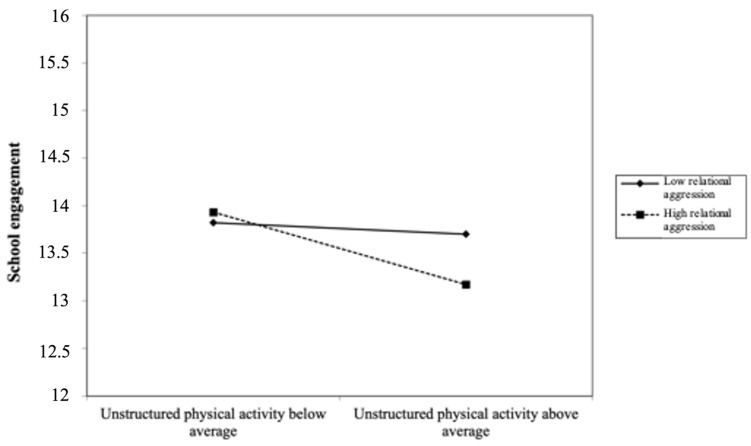
Decomposition of the adjusted interaction between relational aggression and unstructured physical activity at age 6 years associated with school engagement at age 14 years for boys.

Anxiety symptoms also moderated the association between active play and later school engagement in boys. This interaction amplified the negative association between unstructured physical activity at age 6 years and school engagement at age 14 years (β = −0.066, *p* < 0.05, 95% CI: −0.526, −0.021). In other words, boys with high anxiety symptoms who participated in more unstructured physical activity in childhood had substantially lower school engagement years later. Figure 2 shows the decomposition of this interaction. Lastly, relational aggression also moderated the associations between child unstructured physical activity and adolescent school engagement (β = −0.062, *p* < 0.05, 95% CI: −0.614, −0.033). As illustrated in Figure 3, when boys showed high relational aggression in childhood, the greater their involvement in unstructured physical activity at age 6 years, the lower their school engagement at age 14 years.

## 4. Discussion

Active play has decreased in the past years, depriving children of the long-term benefits it fosters [38]. For boys, more active play in childhood was associated with lower subsequent school engagement. This study reveals that socioemotional difficulty moderates the association between active play and later school engagement. It adds to the growing body of research encouraging parents, teachers, and psychosocial professionals to motivate children to choose more active forms of leisure.

Surprisingly, being active without a coach or instructor predicted lower school engagement for boys. This means that boys who were partaking in more physical activity such as biking or skateboarding in childhood were more likely to skip school, not complete their homework, give little importance to their grades and to learning, have little school spirit, and not like school. This could be explained by the fact that boys might be disengaging from school when they are engaging in active play. We are not aware of studies that have shown similar results between physical activity and school engagement. However, Shoval et al. [39] found that boys who were engaging in competitive sports had lower academic achievement than other students. On the other hand, Owen et al. [40] found that implementing a moderate-intensity physical activity session before class improved adolescent cognitive engagement. In a different study, they found that regular physical activity was linked to better engagement in mathematics for boys but not for girls [41]. 

Similarly, we found no direct association between active play and later school engagement for girls. This could stem from the fact that, in early childhood, parents tend to encourage outside play for boys more than for girls, either because of societal norms, lower perceptions of safety or the athletic ability of girls, or because boys tend to ask to play outside more [42]. Girls are also less likely to be urged into sports by their family and peers [43], and they refrain from actively playing when boys are already present [44]. 

These associations were moderated by the child’s socioemotional difficulty. Thus, boys and girls might not be able to fully reap the benefits of an active lifestyle at a young age when they have internalizing or externalizing behavior problems. Girls who were experiencing more anxiety symptoms, like seeming unhappy, crying a lot, or worrying often, in childhood had lower school engagement when they participated in more sports with a coach or an instructor. This could be explained by the fact that girls might be more vulnerable to stress during child development [45]. Young girls, when anxious, may find it more difficult to spread themselves across two domains of performance [46]. Anxiety in children usually affects multiple spheres, including difficulties in school, in social settings, and with their physical health [47]. Boys were experiencing anxiety in a similar way; the more they were active without a coach or instructor, the lower their school engagement was. While they might also find it more difficult to devote effort to two domains at once, they could be more affected by parental biases, as parents tend to favor boys playing freely outside more than they do girls [48]. Moreover, when boys were exhibiting relational aggression, greater participation in physical activities without a coach or instructor resulted in lower subsequent school engagement. This could be explained by the fact that these boys have more difficulties managing their emotions and, therefore, might not thrive in unstructured group settings such as sports [27]. In fact, as a coach or an instructor could serve as a model and a peacekeeper during active play, it is possible that their absence in unstructured physical activity settings would impede the potential benefits of being active for boys with greater behavioral difficulties [15]. Difficulties in managing emotions can also lead to later externalizing problems, especially for boys, which could affect school engagement [21,49].

While these findings are interesting, this study has some methodological challenges. First, as typical in longitudinal research, this study had incomplete data, creating a risk of attrition bias, which was corrected by using multiple imputations. Second, being observational, this study cannot establish causal relationships between active play and later school engagement, although it does set a clear predictor in the time sequence. Third, to address alternate explanations, we included multiple confounding family characteristics as control variables in the analyses. Fourth, the data used are limited to the measures obtained in the realm of a large study on child development. As such, it did not allow us to measure the three dimensions of school engagement (cognitive, emotional, and behavioral) as distinct concepts [32]. It could be that active leisure and socioemotional difficulties are associated with some but not all dimensions of this concept. Finally, active play at age 6 years was reported by the PMK. Our findings are thus limited to the observed instances of active play, which could have been estimated considering that not all parents are with their children all day. Future research should include objective measures of active play.

The primary strength of this study lies in its use of a longitudinal design, tracking participants for nearly a decade and lending temporal context to its findings. This prospective design, coupled with the incorporation of multiple control factors, enabled us to eliminate confounding explanations to our findings. Furthermore, the stratification of the analyses based on sex contributes significant insight to our findings, given the distinctions in how boys and girls engage with sports and schooling. The investigation of the role of socioemotional difficulty in the associations between active play and school engagement adds to the literature on the risk and protective factors in child development. Lastly, the data of the first time point for this study were gathered in 1994, which offers a unique chance to model childhood experiences without the pervasive technological influences present today. While this data may confront challenges in representing how children might choose to occupy their free time with accuracy in our day and age, considering the prominence of technology in the modern context, it does represent a natural experiment in which naturally occurring participation in active play is measured. Hence, our estimates can be deemed both dependable and meaningful. Future research should consider replicating these results with data, taking technological leisure activities into account.

## 5. Conclusions

The increase in sedentary leisure time has become a global concern, as sedentary children face more mental and physical challenges [7]. As lifestyle habits crystallize from a young age, it is paramount to direct children towards active play to allow them to fully reap the benefits of an active lifestyle from preschool onwards. Children with socioemotional difficulties, such as anxiety symptoms, hyperactivity-inattention symptoms, and relational aggression, should be given special consideration to allow them to benefit from active play as much as their counterparts. Future research should continue to highlight the importance of the mutual influence of lifestyle and achievement as a matter of public health [50].

## Figures and Tables

**Table 1:** Descriptive statistics for study variables.

	Boys			Girls		
	M (SD)	Categorical Variables (%)	Range	M (SD)	Categorical Variables (%)	Range
Predictors (age 6 years)						
Organized sport	2.45 (1.32)		1–5	2.11 (1.25)		1–5
1 = almost never		41.5			52.7	
2 = about once a month		3.6			3.2	
3 = about once a week		24.8			24.9	
4 = few times a week		28.6			18.5	
5 = most days		1.5			0.8	
Unstructured physical activity	3.46 (1.54)		1–5	3.11 (1.57)		1–5
1 = almost never		22.3			28.9	
2 = about once a month		4.5			6.0	
3 = about once a week		13.8			16.0	
4 = few times a week		24.1			23.3	
5 = most days		35.4			25.9	
Moderators (age 6 years)						
Hyperactivity-inattention						
1 = symptoms above 1 SD above average		17.5			9.8	
Anxiety						
1 = symptoms above 1 SD above average		19.1			16.9	
Relational aggression						
1 = symptoms above 1 SD above average		13.3			16.5	
Outcome (age 14 years)						
School engagement	13.63 (1.82)		6–18	14.03 (1.87)		6–18
Control variables (age 6 years)						
Family configuration						
1 = non-intact		24.4			24.9	
Income adequacy						
1 = inadequate		2.3			2.8	
Maternal education						
1 = did not graduate high school		18.8			18.6	
Maternal depressive symptoms						
1 = depression symptoms above 1 SD above average		10.7			12.8	
Family dysfunction						
1 = dysfunctional family		9.3			10.5	
Family social support						
1 = social support below average		6.8			6.0	

Notes: Analyses corrected for attrition bias. Data were compiled from the final master file of the National Longitudinal Survey of Children and Youth, Cycles 1–7 (1994–2009), © Statistics Canada.

**Table 2:** Weighted unstandardized regression coefficients (standard error) reflecting the adjusted relationship between socioemotional difficulty and family characteristics at age 6 years and active play at age 6 years for boys and girls.

	Organized Sport		Unstructured Physical Activity	
**Sex**	0.34 (0.04) ***		0.35 (0.05) ***	
	***Boys***	***Girls***	***Boys***	***Girls***
*Socioemotional difficulty*				
Hyperactivity-inattention	−0.15 (0.10)	−0.21 (0.12)	0.03 (0.11)	−0.37 (0.15) *
Anxiety	−0.11 (0.09)	0.11 (0.09)	−0.40 (0.11) ***	0.23 (0.11) *
Relational aggression	0.14 (0.11)	−0.04 (0.09)	0.25 (0.13) *	−0.06 (0.12)
*Family characteristics*				
Family configuration	−0.39 (0.08) ***	−0.33 (008) ***	−0.40 (0.09) ***	0.04 (0.10)
Income adequacy	−0.21 (0.29)	−0.37 (0.26)	0.28 (0.34)	0.01 (0.33)
Maternal education	−0.43 (0.09) ***	−0.37 (0.09) ***	−0.06 (0.10)	0.18 (0.11)
Maternal depressive symptoms	0.19 (0.11)	−0.07 (0.11)	0.09 (0.13)	−0.22 (0.13)
Family dysfunction	−0.20 (0.11)	0.12 (0.11)	−0.23 (0.13)	−0.16 (0.14)
Family social support	−0.45 (0.11) ***	−0.08 (0.12)	−0.51 (0.13) ***	−0.26 (0.15)
R^2^	0.06	0.04	0.05 ***	0.02 *

Notes: * *p* < 0.05, and *** *p* < 0.001. Analyses corrected for attrition bias. Data were compiled from the final master file of the National Longitudinal Survey of Children and Youth, Cycles 1−7 (1994−2009), © Statistics Canada.

**Table 3:** Weighted unstandardized regression coefficients (standard error) reflecting the adjusted relationship between active play, socioemotional difficulty, and family characteristics at age 6 years and school engagement at age 14 years for boys and girls.

	School Engagement	
	*Boys*	*Girls*
Active play variables		
Organized sport	0.03 (0.04)	0.06 (0.04)
Unstructured physical activity	−0.06 (0.03) *	−0.03 (0.03)
Socioemotional difficulty		
Hyperactivity-inattention	−0.13 (0.13)	0.09 (0.17)
Anxiety	0.14 (0.12)	−0.35 (0.13) **
Relational aggression	−0.21 (0.14)	0.25 (0.13)
Interactions		
Organized sport × Hyperactivity-inattention	−0.11 (0.13)	0.12 (0.17)
Organized sport × Anxiety	−0.03 (0.12)	−0.28 (0.13) *
Organized sport × Relational aggression	0.06 (0.14)	0.07 (0.13)
Unstructured physical activity × Hyperactivity-inattention	−0.18 (0.13)	0.17 (0.17)
Unstructured physical activity × Anxiety	−0.27 (0.13) *	0.25 (0.13)
Unstructured physical activity × Relational aggression	−0.32 (0.15) *	0.09 (0.15)
Family characteristics		
Family configuration	−0.21 (0.11) *	−0.51 (0.12) ***
Income adequacy	−0.06 (0.39)	0.28 (0.37)
Maternal education	−0.02 (0.12)	−0.38 (0.13) **
Maternal depressive symptoms	0.02 (0.15)	0.15 (0.16)
Family dysfunction	0.05 (0.15)	−0.35 (0.16) *
Family social support	0.40 (0.15) **	−0.32 (0.18)
R^2^	0.01 *	0.04 ***

Notes: * *p* < 0.05, ** *p* < 0.01, and *** *p* < 0.001. Analyses corrected for attrition bias. Data were compiled from the final master file of the National Longitudinal Survey of Children and Youth, Cycles 1−7 (1994−2009), © Statistics Canada.

## Data Availability

The data presented in this study are available on request from Statistics Canada. The data are not publicly available due to permission from Statistics Canada.

## Appendix A. Measures

### Appendix A.1. Socioemotional Moderation Variables (Age 6 Years)

*Hyperactive-inattentive behavior.* The PMKs completed an 8-item scale (α = 0.84) regarding hyperactive and inattentive behavior. They were asked about their child’s behavior: “How often would you say that your child: Can’t sit still, is restless or hyperactive?; Is distractible, has trouble sticking to any activity?; Fidgets?; Can’t concentrate, can’t pay attention for long?; Is impulsive, acts without thinking?; Has difficulty awaiting turn in games or groups?; Cannot settle to anything for more than a few moments?; Is inattentive?”. The responses ranged from 1 = Never or not true, 2 = Sometimes or somewhat true, and 3 = Often or very true. Out of those questions, six were taken from the Ontario Child Health Study, and one was taken from the Montreal Longitudinal and Experimental Study. The scores were added to create a scale where a higher score indicates a more hyperactive and inattentive child. The scores were computed as 0 = below one standard deviation above the mean and 1 = above one standard deviation above the mean/at risk.

*Anxious behavior.* The PMKs completed an 8-item scale (α = 0.79) regarding anxious behavior. They were asked about their child’s behavior: “How often would you say that your child: Seems to be unhappy, sad or depressed?; Is not as happy as other children?; Is too fearful or anxious?; Is worried?; Cries a lot?; Appears miserable, unhappy, tearful, or distressed?; Is nervous, highstrung or tense?; Has trouble enjoying him/herself?”. Out of those questions, four were taken from the Ontario Child Health Study, and two were taken from the Montreal Longitudinal and Experimental Study. The responses ranged from 1 = Never or not true, 2 = Sometimes or somewhat true, and 3 = Often or very true. The scores were added to create a scale where a higher score indicates a more anxious child. The scores were computed as 0 = below one standard deviation above the mean and 1 = above one standard deviation above the mean/at risk.

*Relational aggression.* The PMKs completed a 5-item scale (α = 0.78) regarding relational aggressive behavior. They were asked about their child’s behavior: “How often would you say that your child: When mad at someone, tries to get others to dislike that person?; When mad at someone, becomes friends with another as revenge?; When mad at someone, says bad things behind the other’s back?; When mad at someone, says to others: let’s not be with him/her?; When mad at someone, tells the other one’s secrets to a third person?”. Those questions were taken from the Direct and Indirect Aggression Scale (DIAS) by Björkqvist et al. [31]. The responses ranged from 1 = Never or not true, 2 = Sometimes or somewhat true, and 3 = Often or very true. The scores were added to create a scale where a higher score indicates more relational aggression by a child. The scores were computed as 0 = below one standard deviation above the mean and 1 = above one standard deviation above the mean/at risk.

### Appendix A.2. School Engagement Outcomes (Age 14 Years)

The youth participants completed a 6-item scale on behavioral, affective, and cognitive aspects of school engagement (α = 0.71 for girls; α = 0.68 for boys [32]). Each item was recalculated on a scale from 1 to 3, and a scale ranging from 6 (very low school engagement) to 18 (very high school engagement) was formed from the sum of the scores.

*Behavioral engagement.* The youth were asked: “In the past 12 months, how many times have you skipped a day of school without permission?” (1 = 5 times or more, 2 = 3 or 4 times, 3 = Once or twice, or 4 = Never) and “When my teachers give me homework, I do it.” (1 = Never, 2 = Rarely, 3 = Some of the time, 4 = Most of the time, or 5 = All the time).

*Cognitive engagement.* The youth were asked: “How important is it to you to do the following in school: (a) Get good grades? and (b) Learn new things?” (1 = Not important at all/Not very important, 2 = Somewhat important, or 3 = Very important).

*Affective engagement.* The youth were asked: “How do you feel about school?” (1 = I hate school, 2 = I don’t like school very much, 3 = I like school a bit, 4 = I like school quite a bit, or 5 = I like school very much), and “How much school spirit do you have?” (1 = None, 2 = Very little, 3 = Some, or 4 = A great deal).

### Appendix A.3. Measures: Family Control Variables (Age 6 Years)

*Family configuration* was derived from basic demographic information. A family is considered intact when the couple is either married or common-law and where children are the natural and/or adopted offspring of both members of the couple (0 = intact family and 1 = non-intact family/at risk).

*Income adequacy* was computed from the household income reported by the PMKs into five following categories: lowest, lower-middle, middle, upper-middle, and highest (0 = lower-middle, middle, upper-middle, or highest, and 1 = lowest/at risk).

*Maternal education* was reported by the PMKs. They were asked: “Have you graduated from high school?” (0 = graduated high school and 1 = did not graduate high school/at risk).

*Maternal depressive symptoms* were reported by the PMKs and assessed with a 12-item abridged version of the original 20-item CES-D depression rating scale ([33], α = 0.82). They were asked, “How often have you felt or behaved this way during the past week: I did not feel like eating, my appetite was poor; I felt that I could not shake off the blues even with help from my family or friends; I had trouble keeping my mind on what I was doing; I felt depressed; I felt that everything I did was an effort; I felt hopeful about the future; My sleep was restless; I was happy; and How often have you felt or behaved this way during the past week: I felt lonely; I enjoyed life; I had crying spells; I felt that people disliked me.” The responses ranged from 1 = Rarely or none of the time (less than 1 day); 2 = Some or a little of the time (1–2 days); 3 = Occasionally or a moderate amount of the time (3–4 days); 4 = Most or all of the time (5–7 days). The final scores ranged from 0 to 36, where a higher score indicates the presence of more depressive symptoms. The scores were computed as 0 = below one standard deviation above the mean and 1 = above one standard deviation above the mean/at risk.

*Family dysfunction* was assessed using the 12-item McMaster Family Assessment Device ([34], α = 0.88). The PMKs were asked: “The following statements are about families and family relationships. For each one, please indicate which response best describes your family: Planning family activities is difficult because we misunderstand each other; In times of crisis we can turn to each other for support; We cannot talk to each other about sadness we feel; Individuals (in the family) are accepted for what they are; We avoid discussing our fears or concerns; We express feelings to each other; There are lots of bad feelings in our family; We feel accepted for what we are; Making decisions is a problem for our family; We are able to make decisions about how to solve problems; We don’t get along well together; We confide in each other.” The responses ranged from 1 = Stongly agree; 2 = Agree; 3 = Disagree; 4 = Strongly disagree. The final scores ranged from 0 to 36, where a higher score indicates dysfunction in problem-solving and communication within the family. The scores were computed as 0 = below one standard deviation above the mean and 1 = above one standard deviation above the mean/at risk.

*Family social support* was assessed only at Cycle 1 and Cycle 3 using a 6-item scale (α = 0.88) derived from the 24 items in the Social Provisions Model [35]. The PMKs were asked: “If something went wrong, no one would help me; I have family and friends who help me feel safe, secure and happy; There is someone I trust whom I would turn to for advice if I were having problems; There is no one I feel comfortable talking about problems with; I lack a feeling of closeness with another person; There are people I can count on in an emergency”. The responses ranged from 1 = Strongly disagree; 2 = Disagree; 3 = Agree; 4 = Strongly agree. The final scores ranged from 0 to 24, with higher scores indicating the presence of social support in the family. The scores were computed as 0 = above one standard deviation below the mean and 1 = below one standard deviation below the mean/at risk.

## References

[1] Lioret S., Campbell K.J., McNaughton S.A., Cameron A.J., Salmon J., Abbott G., Hesketh K.D. (2020). Lifestyle patterns begin in early childhood, persist and are socioeconomically patterned, confirming the importance of early life interventions. Nutrients.

[2] de Lannoy L., Rhodes R.E., Moore S.A., Faulkner G., Tremblay M.S. (2020). Regional differences in access to the outdoors and outdoor play of Canadian children and youth during the COVID-19 outbreak. Can. J. Public Health.

[3] Moore S.A., Faulkner G., Rhodes R.E., Brussoni M., Chulak-Bozzer T., Ferguson L.J., Mitra R., O’Reilly N., Spence J.C., Vanderloo L.M. (2020). Impact of the COVID-19 virus outbreak on movement and play behaviours of Canadian children and youth: A national survey. Int. J. Behav. Nutr. Phys. Act..

[4] Parent N., Guhn M., Brussoni M., Almas A., Oberle E. (2021). Social determinants of playing outdoors in the neighbourhood: Family characteristics, trust in neighbours and daily outdoor play in early childhood. Can. J. Public Health.

[5] Tandon P.S., Hafferty K., Kroshus E., Angulo A., Burton M., Peyton M., Senturia K. (2022). A framework for pediatric health care providers to promote active play in nature for children. J. Prim. Care Community Health.

[6] Lee E.Y., Bains A., Hunter S., Ament A., Brazo-Sayavera J., Carson V., Hakimi S., Huang W.Y., Janssen I., Lee M. (2021). Systematic review of the correlates of outdoor play and time among children aged 3–12 years. Int. J. Behav. Nutr. Phys. Act..

[7] Carson V., Chaput J.P., Janssen I., Tremblay M.S. (2017). Health associations with meeting new 24-hour movement guidelines for Canadian children and youth. Prev. Med..

[8] Truelove S., Vanderloo L.M., Tucker P. (2017). Defining and measuring active play among young children: A systematic review. J. Phys. Act. Health.

[9] Moghaddaszadeh A., Belcastro A.N. (2021). Guided active play promotes physical activity and improves fundamental motor skills for school-aged children. J. Sports Sci. Med..

[10] Janssen I. (2014). Active play: An important physical activity strategy in the fight against childhood obesity. Can. J. Public Health.

[11] Leonard H.C., Hill E.L. (2014). Review: The impact of motor development on typical and atypical social cognition and language: A systematic review. Child. Adolesc. Ment. Health.

[12] Owen K.B., Parker P.D., Van Zanden B., MacMillan F., Astell-Burt T., Lonsdale C. (2016). Physical activity and school engagement in youth: A systematic review and meta-analysis. Educ. Psychol..

[13] Tam K.I., Philpott-Robinson K., Johnson T., Lane A.E. (2023). Measurement of school engagement in elementary school students: A scoping review. Am. J. Occup. Ther..

[14] Lerner R.M., Lerner J.V., Geldhof G.J., Gestsdóttir S., King P.E., Sim A.T., Dowling E., Lansfrord J.E., Banati P. (2018). Studying Positive Youth Development in Different Nations: Theoretical and Methodological Issues. Handbook of Adolescent Development Research and Its Impact on Global Policy.

[15] Holt N.L., Deal C.J., Pankow K., Tenenbaum G., Eklund R.C. (2020). Positive youth development through sport. Handbook of Sport Psychology.

[16] Martinot D., Sicard A., Gul B., Yakimova S., Taillandier-Schmitt A., Maintenant C. (2022). Peers and teachers as the best source of social support for school engagement for both advantaged and priority education area students. Front. Psychol..

[17] Zhao Y., Zheng Z., Pan C., Zhou L. (2021). Self-esteem and academic engagement among adolescents: A moderated mediation model. Front. Psychol..

[18] Pérez-Salas C.P., Parra V., Sáez-Delgado F., Olivares H. (2021). Influence of teacher-student relationships and special educational needs on student engagement and disengagement: A correlational study. Front. Psychol..

[19] Sleddens E.F.C., Gubbels J.S., Kremers S.P.J., van der Plas E., Thijs C. (2017). Bidirectional associations between activity-related parenting practices, and child physical activity, sedentary screen-based behavior and body mass index: A longitudinal analysis. Int. J. Behav. Nutr. Phys. Act..

[20] Neudecker C., Mewes N., Reimers A.K., Woll A. (2019). Exercise interventions in children and adolescents with ADHD: A systematic review. J. Atten. Disord..

[21] Rushton S., Giallo R., Efron D. (2020). ADHD and emotional engagement with school in the primary years: Investigating the role of student-teacher relationships. Brit. J. Educ. Psychol..

[22] Rodriguez-Ayllon M., Cadenas-Sánchez C., Estévez-López F., Muñoz N.E., Mora-Gonzalez J., Migueles J.H., Molina-García P., Henriksson H., Mena-Molina A., Martínez-Vizcaíno V. (2019). Role of physical activity and sedentary behavior in the mental health of preschoolers, children and adolescents: A systematic review and meta-analysis. Sports Med..

[23] Gunnell K.E., Flament M.F., Buchholz A., Henderson K.A., Obeid N., Schubert N., Goldfield G.S. (2016). Examining the bidirectional relationship between physical activity, screen time, and symptoms of anxiety and depression over time during adolescence. Prev. Med..

[24] Bélair M.A., Kohen D.E., Kingsbury M., Colman I. (2018). Relationship between leisure time physical activity, sedentary behaviour and symptoms of depression and anxiety: Evidence from a population-based sample of Canadian adolescents. BMJ Open.

[25] Olivier E., Morin A.J.S., Langlois J., Tardif-Grenier K., Archambault I. (2020). Internalizing and externalizing behavior problems and student engagement in elementary and secondary school students. J. Youth Adolesc..

[26] Swit C.S., Harty S.C., Pascoe S. (2024). Relational and physical aggression in preschool-age children: Associations with teacher, parent, sibling, and peer relationship quality. Aggress. Behav..

[27] Ersan C. (2020). Physical aggression, relational aggression and anger in preschool children: The mediating role of emotion regulation. J. Gen. Psychol..

[28] Hinkley T., Brown H., Carson V., Teychenne M. (2018). Cross sectional associations of screen time and outdoor play with social skills in preschool children. PLoS ONE.

[29] Wu W.C., Chang L.Y., Luh D.L., Wu C.C., Stanaway F., Yen L.L., Chang H.Y. (2020). Sex differences in the trajectories of and factors related to extracurricular sport participation and exercise: A cohort study spanning 13 years. BMC Public Health.

[30] Lietaert S., Roorda D., Laevers F., Verschueren K., De Fraine B. (2015). The gender gap in student engagement: The role of teachers’ autonomy support, structure, and involvement. Brit. J. Educ. Psychol..

[31] Björkqvist K., Österman K., Kaukiainen A., Björkqvist K., Niemelä P. (1992). The development of direct and indirect aggressive strategies in males and females. Of Mice and Women: Aspects of Female Aggression.

[32] Wang M.T., Fredricks J.A. (2014). The reciprocal links between school engagement, youth problem behaviors, and school dropout during adolescence. Child. Dev..

[33] Radloff L.S. (1977). The CES-D Scale: A self-report depression scale for research in the general population. Appl. Psychol. Meas..

[34] Epstein N.B., Baldwin L.M., Bishop D.S. (1983). The McMaster Family Assessment Device. J. Marital. Fam. Ther..

[35] Weiss R., Rubin Z. (1974). The provisions of social relationships. Doing unto Others.

[36] Aiken L.S., West S.G. (1991). Multiple Regression: Testing and Interpreting Interactions.

[37] Dawson J.F. (2014). Moderation in management research: What, why, when and how. J. Bus. Psychol..

[38] Auhuber L., Vogel M., Grafe N., Kiess W., Poulain T. (2019). Leisure activities of healthy children and adolescents. Int. J. Environ. Res. Public Health.

[39] Shoval E., Shachaf M., Ramati-Dvir O., Shulruf B. (2021). Gender matters when sports engagement and self-efficacy interact with academic achievement. Soc. Psychol. Educ..

[40] Owen K.B., Parker P.D., Astell-Burt T., Lonsdale C. (2018). Effects of physical activity and breaks on mathematics engagement in adolescents. J. Sci. Med. Sport.

[41] Owen K.B., Parker P.D., Astell-Burt T., Lonsdale C. (2018). Regular physical activity and educational outcomes in youth: A longitudinal study. J. Adolesc. Health.

[42] Visser K., van Aalst I. (2022). Neighbourhood Factors in Children’s Outdoor Play: A Systematic Literature Review. J. Econ. Hum. Geogr..

[43] Telford R.M., Telford R.D., Olive L.S., Cochrane T., Davey R. (2016). Why are girls less physically active than boys? Findings from the LOOK longitudinal study. PLoS ONE.

[44] Reimers A.K., Schoeppe S., Demetriou Y., Knapp G. (2018). Physical activity and outdoor play of children in public playgrounds – Do gender and social environment matter?. Int. J. Environ. Res. Public Health.

[45] Hodes G.E., Epperson C.N. (2019). Sex differences in vulnerability and resilience to stress across the life span. Biol. Psychiatry.

[46] Strömbäck M., Wiklund M., Renberg E.S., Malmgren-Olsson E.B. (2015). Complex symptomatology among young women who present with stress-related problems. Scand. J. Caring Sci..

[47] Doyle M.M. (2022). Anxiety disorders in children. Pediatr. Rev..

[48] Boxberger K., Reimers A.K. (2019). Parental correlates of outdoor play in boys and girls aged 0 to 12—A systematic review. Int. J. Environ. Res. Public Health.

[49] Behrendt H.F., Wade M., Bayet L., Nelson C.A., Bosquet Enlow M. (2020). Pathways to social-emotional functioning in the preschool period: The role of child temperament and maternal anxiety in boys and girls. Dev. Psychopathol..

[50] Paasche-Orlow M.K., Wolf M.S. (2010). Promoting health literacy research to reduce health disparities. J. Health Commun..

